# Health Research Ethics Committees in South Africa 12 years into democracy

**DOI:** 10.1186/1472-6939-8-1

**Published:** 2007-01-25

**Authors:** Keymanthri Moodley, Landon Myer

**Affiliations:** 1 Associate Professor, Bioethics Unit-Tygerberg Division, Centre for Applied Ethics & Faculty of Health Sciences, University of Stellenbosch, South Africa; 2 Senior Lecturer, Infectious Diseases Epidemiology Unit, School of Public Health & Family Medicine, University of Cape Town, South Africa

## Abstract

**Background:**

Despite the growth of biomedical research in South Africa, there are few insights into the operation of Research Ethics Committees (RECs) in this setting. We investigated the composition, operations and training needs of health RECs in South Africa against the backdrop of national and international guidelines.

**Methods:**

The 12 major health RECs in South Africa were surveyed using semi-structured questionnaires that investigated the composition and functions of each REC as well as the operational issues facing committees.

**Results:**

Health RECs in SA have an average of 16 members and REC members are predominantly male and white. Overall, there was a large discrepancy in findings between under-resourced RECs and well resourced RECs. The majority of members (56%) are scientists or clinicians who are typically affiliated to the same institution as the health REC. Community representatives account for only 8% of membership. Training needs for health REC members varied widely.

**Conclusion:**

Most major health RECs in South Africa are well organized given the resource constraints that exist in relation to research ethics in developing countries. However, the gender, racial and occupational diversity of most of these RECs is suboptimal, and most RECs are not constituted in accordance with South African guidelines. Variability in the operations and training needs of RECs is a reflection of apartheid-entrenched influences in tertiary education in SA. While legislation now exists to enforce standardization of research ethics review systems, no provision has been made for resources or capacity development, especially to support historically-disadvantaged institutions. Perpetuation of this legacy of apartheid represents a violation of the principles of justice and equity.

## Background

The system of ethical review in South Africa dates back to 1966 when the first REC was established at the University of the Witwatersrand, and since then most major tertiary institutions have developed research ethics committees. Today there are approximately 34 local RECs in South Africa [1], two of which are part of private, non-academic institutions. In 2002, the Office for Human Research Protections (OHRP) visited SA and granted Federal Wide Assurances (FWAs) to 8 RECs in the country. The FWAs were part of the OHRP's Quality Assurance process whereby written policies and procedures were compared to practice. This is a voluntary process and the FWA is an endorsement of an institution's commitment to such quality assurance. Recently, the National Health Act [2] has made provision for the establishment of a National Health Research Ethics Council (NHREC) whose responsibility it is to set guidelines for the functioning of local RECs, to register and audit local RECs, to set norms and standards for research, to adjudicate complaints about the functioning of RECs and to institute disciplinary action against those who violate norms or guidelines for research in terms of the Act.

In 2000, the South African Good Clinical Practice (GCP) guideline was published by the Department of Health [3]. This national guideline specifies composition of RECs in terms of race, gender and occupational identity. A more recent guideline released by the Department of Health has revised and strengthened these requirements [1]. However, there are no data to document whether RECs in SA are constituted in accordance with these national guidelines.

In 2001, the World Health Organisation Regional Committee for Africa expressed concern that some health related studies undertaken in the region were not subjected to any form of ethics review [4]. While the practice of ethical review by health RECs is firmly entrenched in South Africa [5], the quality and consistency of ethical review in South Africa remains unclear. There are few insights into the capacity of RECs in South Africa. South Africa is a popular research site for multinational collaborative research[6] and hence is involved in dual review of hundreds of research protocols annually [7]. Some of the reasons quoted for conducting research in Africa rather than in developed countries include lower costs, lower risk of litigation and less stringent ethical review [8]. Concerns have also been raised that RECs in developing countries may not promote high standards of research participant protection as a result of a lack of financial and adequately trained human resources [9]. Hence, data on developing country RECs are of interest to researchers and RECs around the world especially those who undertake research in South Africa.

Given the explosion of biomedical research involving human participants in South Africa, and the lack of insight into research ethics review infrastructure, we examined the composition, operations and training needs of the major health RECs in South Africa against the backdrop of national and international guidelines.

## Methods

The RECs chosen for this survey were identified in 2003 via the chair of the Interim National Health Research Ethics Committee who provided a list of 22 RECs. Of these three were private and 19 institutional. Nine of the 19 were attached to universities: University of Cape Town, University of Stellenbosch, University of Pretoria, Medical University of South Africa, University of Witwatersrand, University of Transkei, University of Kwa-Zulu Natal, University of Orange Free State. The ninth REC is attached to the Medical Research Council (MRC) of South Africa. The remaining 10 academic RECs are smaller, part-time committees attached to Technicons (vocational colleges). These RECs were excluded from this sample due to a transition process involving mergers of various institutions in keeping with a rationalisation process in higher education. If a committee was involved with very little health research, if any, it was also excluded. Where RECs were attached to universities, only those in the Health Sciences Faculties were included in the sample as this is where health research is primarily reviewed.

Approval for the study was granted by the Research Committee, University of Stellenbosch. The study was conducted in accordance with the Declaration of Helsinki 2000 [10]. Permission to administer the questionnaires to administrative officers and to conduct the interviews with chairpersons was sought telephonically from the Chairperson of each REC. All 12 REC chairpersons agreed to participate and appointments were secured at the various institutions. Chairpersons were assured that the data obtained would not be linked to specific RECs. After the interviews were conducted all data was anonymised. The quantitative component of the study assumed the format of a descriptive survey of RECs in South Africa that was based on a structured questionnaire. This method ensured that each REC was exposed to the same questions so that their responses could be reliably compared [11]. Development of the research tools (questionnaire and interview guide) was based on the basic functions that are intrinsic to any research ethics review system [12] as well as known deficiencies in the system including REC membership, education and training of REC members, lack of institutional commitment, inadequate initial and continuing review of protocols, informed consent and review of research on vulnerable populations.

The questionnaire collected information on membership, workload, efficiency, review procedures, infrastructure and resources. It was administered to the administrative officer of each of 12 RECs in South Africa during 2003. The questionnaire was completed with the assistance of the interviewer. This method of face-to-face completion of structured questionnaires enabled the form to be completed comprehensively[11]. It also ensured a better response rate compared to self-administered questionnaires that are usually used in a postal survey. When the administrative officer was unable to respond to any of the questions these answers were obtained from the chairperson of the REC.

## Results

### General

Twelve health research ethics committees participated in the quantitative survey: Nine institutional committees associated with University Faculties of Health Sciences that review academic research and industry-sponsored trials, as well as three private committees (Pharmaethics, South African Medical Association, and Anglogold) that review primarily industry-sponsored studies. The size of the 12 committees ranged from seven to 29 members (median, 16 members), excluding alternate members. Of the 12 committees, ten had been in operation for at least ten years, with the oldest established more than 30 years previously. The remaining two committees were less than a year old at the time of the survey and both were linked to historically disadvantaged academic institutions.

### Diversity

All of the committees, except one, were comprised of mostly men (mean proportion men, 63%, range 46%–82%). Amongst REC members, the representation of women ranged from 18% to 54%. In 83% of health RECs surveyed, less than half the members were female. All but three committees were comprised of mostly white members (mean proportion white, 62%, range 10%–86%).

Most of the committees were comprised primarily of health scientists/clinicians, (56% of all committee members) – see table 1. At institutions, the RECs are larger, hence a variety of scientific disciplines are represented on the committee. The private RECs are much smaller than institutional RECs and have very limited scientific review capacity – one REC had only one scientific/clinical member.

**Table 1:** Ethics committee composition in terms of profession

Membership category	Category representation on the 12 RECs	Percentage of total committee membership in SA
Health scientists/clinicians	12	56%
Nurses	9	6%
Pharmacists	5	5%
Allied health professionals	5	5%
Lawyers	9	8%
Ethicists	8	6%
Theologians(Christian)	9	5%
Community representatives	10	8%
Statisticians	3	1%

Community representatives are defined as people who are not involved in medical, legal and/or scientific work and who are preferably from the community [3]. Of the ten committees reporting a "community representative", the type of representatives included: priests, a theologian with a doctoral degree, an ethicist with a doctoral degree, union members, educators (including a retired headmaster), local business people, students, a retired nurse, and a Non Governmental Organisation (NGO) member. While most of these people were not involved in medical, legal or scientific work, they were not necessarily from the communities being researched. The RECs could substantiate that these individuals qualified as community representatives as they complied with the SAGCP guideline (2000) definition which states that these representatives should "preferably" be from the community.

Two of the committees reported that all their members were independent or not affiliated with the institution, while another two reported that none of their members were independent of the institution. The remaining eight committees reported that less than 50% of their members were independent or non-affiliated.

### Training and development of REC members in research ethics

There is a wide differential between the different institutions in terms of training of REC members. While at two institutions all REC members have received training in research ethics, at other institutions none of the members had been trained at the time of the interview. Most of the training had been attendance of Good Clinical Practice workshops. Some RECs provide in-house training, time permitting, at meetings. Others circulate articles on Research Ethics to members. Funding of RECs is a perpetual problem at most institutions as these bodies receive low priority within institutions. Many RECs indicated that funding for training is available as a result of fees charged for protocol review. This charge is levied only for sponsored research. No charge is levied for non-sponsored investigator driven research. Institutions with the lowest training levels are either not charging for review of sponsored research or are charging much less than the other RECs.

### Workload

The average number of protocols reviewed per meeting varied from four to 30, with a mean of 12. This included both clinical trials and academic research for all RECs except one that processes only clinical trials while academic research is processed by a separate REC altogether. This is the only institution in South Africa that has three different RECs, one for clinical trials, one for human research (academic) and one for animal research.

The median estimated number of protocols reviewed during 2002 was 135, with a range from 30 to 360 (the total number of protocols reviewed during 2002 by the 12 committees was estimated at over 1600). Six RECs had data for the number of protocols rejected during 2002. The rejection percentage for these six committees ranged from 0% to 10% with a mean of 4,52%.

### Efficiency of protocol review time

The average time from protocol submission to response was five weeks, with a range from ten days to ten weeks. The REC that meets fortnightly is able to process reviews within the shortest time possible. However, this is a private REC and has more fulltime members and members from the private sector who do not have the same workload as academics working at universities. One-third of the committees (four of twelve) reported no charge for submissions; of the remaining eight that did charge investigators submitting a protocol, the average cost was approximately R2700 ($415) (range, R2000 to R5500/$308 – $846). This income was essential to the functioning of RECs as most institutions typically fund office space and one administrative assistant post only. All other REC work is conducted on a voluntary basis by committee members who derive their income from other disciplines.

### Infrastructure and administrative staff

Nine of the 12 committees reported having an office dedicated to the committee's activities. One committee reported having no staff dedicated to the committee's operations. Of the remaining 11 committees, most (n = 9) had at least one full-time staff member, with three committees reporting three full-time staff members.

## Discussion

In general, most participating South African health RECs have been established for at least the last 10 years, are reviewing a mean of 12 protocols per meeting and are providing a response to investigators at an average time of 5 weeks. Two important features are highlighted in this study – firstly, the inadequacy of diversity of REC membership in SA and secondly, the variability in operations, infrastructure and training needs amongst the various RECs.

In 2005, the Department of Health released a national guideline outlining ethics in health research in South Africa [1]. Regarding diversity, section 4.1 requires that RECs must be "representative of the communities they serve and increasingly reflect the demographic profile of the population of South Africa; have a minimum membership of at least nine members, with 60% constituting a quorum". The committee should "include members of both gender and not more than 70% of its members must be men or women". With respect to occupational identity, there must be "at least two lay persons with no affiliations with the institution, not currently involved in medical, scientific or legal work and who are preferably from the community in which research is to take place; at least one member with knowledge of and current experience in areas of research that are regularly considered by the ethics committee; at least one member with knowledge of, and current experience in the professional care, counselling or treatment of people(e.g. medical practitioner, psychologist, social worker, nurse); and at least one member who is legally trained. In addition, there should be "at least one member who has professional training in both qualitative and quantitative research methodologies".

These guidelines are similar, in principle, to the International Conference of Harmonisation Harmonised Tripartite guideline for Good Clinical Practice (ICH GCP) [13], except for minimum membership of five in the international guideline. In addition, given our history of racial and gender discrimination, the requirements for diversity, demographic representation and occupational identity are specified in more detail in the South African guidelines.

This survey indicates that most committees exceed current minimum membership requirements in terms of numbers. However, composition in terms of gender and race does not meet South African requirements. In most instances the lack of diversity on RECs is attributed to the university faculty community – most of whom are white males. The paucity of Black faculty members at most health sciences faculties in South Africa is one of the many consequences of South Africa's history of racial discrimination and the impact it had on the training of Black medical students [14]. Blacks, in particular those classified as African under the apartheid system, were restricted entry into medical schools by a permit system in operation from 1959 to 1986 [15]. Twelve years after democracy, while SA has made political progress at a national and international level, health sciences and health science research in particular have lagged behind in achieving equity. This may be an indicator of resistance to change within the health sciences profession.

Diversity is an important consideration in SA given the political history and the asymmetrical power relationship between researchers (who are predominantly white) and participants (who tend to be people of colour). Undoubtedly, SA is home to many vulnerable groups of poor communities who have limited or no access to education and health services and who accept authority without question [1,3]. Many research participants and research communities meet the definition for vulnerability outlined by the joint United Nations Program on HIV/AIDS (UNAIDS) : limited economic development; inadequate protection of human rights and discrimination on the basis of health status; inadequate community/cultural experience with the understanding of scientific research; limited availability of health care and treatment options and limited ability of individuals in the community to provide informed consent [16]. In the past in SA, the rights of such communities have been violated by health care providers in positions of authority [17]. These violations have also occurred in the context of research. The breast cancer study conducted by Werner Bezwoda, a white oncology/haematology professor, on black women at Baragwanath hospital in South Africa in 1995, without consent or ethics review, placed the health and lives of these women at considerable risk [18]. The international precedent for such research ethics violations had been set by white American researchers in the Tuskegee study in 1932 where poor African-American males were enrolled into a Syphilis study that was riddled with human rights violations[19]. As a result, when HIV research escalated from 1997 onwards and predominantly white researchers were enrolling predominantly black participants in developing countries, parallels were drawn with Tuskegee [20]. Empirical research to assess the impact of Tuskegee on perceptions of African-Americans in relation to research has demonstrated a negative impact on research to the extent that distrust of researchers poses a substantial barrier to recruitment for all types of research [21-23]. Historically, both in SA and in the United States, the conduct of research by white researchers on people of colour has been ethically challenging and controversial.

The primary duty of a REC is participant protection. In addition the South African guideline specifies composition of such a committee in terms of race, gender and occupational identity. The OHRP echoes this requirement in its Code of Federal Regulations (CFR) part 46.107: "The IRB shall be sufficiently qualified though the experience and expertise of its members, and the diversity of the members, including consideration of race, gender and cultural backgrounds and sensitivity to such issues as community attitudes, to promote respect for its advice and counsel in safeguarding the rights and welfare of human subjects"[24]. It may be argued that participant protection by an REC can occur irrespective of diversity in REC membership. However, in SA, where the rights of vulnerable people of colour have been violated in the past by government structures composed mainly of white males, participant protection must be entrusted to a demographically representative REC in order "to promote respect for its advice and counsel"[24]. Hence in SA it is important that protection of the rights of study participants is entrusted to a representative group of people. The composition of RECs as predominant white male committees could be perceived as reinforcing the asymmetrical power relationship that already exists between predominantly white researchers and predominantly black participants. This perception needs to be demonstrated in SA with empirical research.

Diversity in gender distribution is also problematic. Except for one institution, all Chairpersons of RECs in South Africa are male. This parallels the gender disparities that exist in academia with very few women occupying senior faculty positions. Three RECs have male representation in excess of the 70% specified by the guideline from the Department of Health [1,3].

While most committees reflect professional diversity in membership, there is a dominance of health scientists/clinicians. Amongst the 12 South African RECs surveyed, doctors, scientists and pharmacists together made up 61% of the membership. This exceeds international scientific/clinical membership trends. A survey of 89 IRBs in the United States in 2001 found that physicians, scientists and pharmacists together made up 46% of IRB membership [25].

While scientific/clinical membership is high on most RECs less than half of all RECs have a pharmacologist as a scientific/clinical member, yet all RECs in South Africa review both the science and the ethics of protocols. This represents a serious hiatus as almost all RECs included in the survey are involved in the review of clinical trials of investigational drugs. Unlike many other countries in Africa [4], ethicists are well represented on South African RECs, in keeping with international trends [25].

Lay representation is present in 80% of RECs but these are not always people from the community. A distinction needs to be drawn between lay representation and community representation. Lay representation on a research ethics committee usually refers to anyone who has no scientific or medical background. It could therefore include lawyers, ethicists, priests or theologians. The National Bioethics Advisory Commission (NBAC, 2001) in the United States recommends that non-scientists make up at least 25 % of an IRB's membership [26]. In South Africa, these "lay" members are in the minority on all RECs and are not always members of the community being researched. They have a higher level of education than lay community members and while they play an important role in lending a multidisciplinary approach to the review process, they are not ideally suited to assess the patient information leaflets from the perspective of a community member. Community representatives, on the other hand, would refer to non-professional, non-scientific members who belong to the community that is being researched. Generally they would be conversant in the same language as the research participants and they would share a similar culture.

In most RECs surveyed in South Africa there is blurring of these two different categories of representatives. This is reinforced by the South African research ethics guidelines which conflate lay representation with community representation [1,3]. Often the lay person is taken to be the lawyer or priest with much higher levels of education than the average of the community being researched. Often the priest is someone of the Christian faith with lack of representation of other religious sects in the community. The requirement of the guidelines for lay members to "preferably" be from communities in which research is conducted does not adequately support the important role that community members play on a REC [27].

Given the large number of vulnerable populations involved in health research in SA, efforts to promote community representation on health RECs and in research must be encouraged. This can be achieved by ensuring that REC guidelines on membership distinguish between lay representation and community representation. By specifying the proportion of membership that should account for both categories, for example, 25% lay members and 25% community members, balance and objectivity will be added to most RECs especially those that are predominantly composed of scientists and clinicians affiliated to institutions where the proposed research under review is to be conducted. Specifying that a quarter of the REC should comprise community members (as opposed to 1 or 2 members) prevents the intimidation that community members often experience in an REC that is top heavy in terms of scientists or clinicians and obviates the power differentials that would prevent adequate and fair participation of community members [27]. Finally, education of community representatives in research ethics is to be encouraged as this would empower them to deliberate issues that impact on participant protection in a more meaningful way.

Most members of institutional health RECs in South Africa are affiliated to the institution. This parallels trends internationally [25]. Where the majority of members are affiliated to the institution, the potential for biased and inadequate review exists. Members with no affiliation to the institution are important to lend objectivity to the review process and protect the REC from facing a conflict of interest in reviewing research that will benefit the institution.

As a result of the large proportion of scientific and clinical members on RECs in South Africa it is inevitable that some members play dual roles of investigators and REC members and submit their protocols to their own institutional REC. It is standard practice that the member may not contribute to the discussion (except for responding to queries raised during the review) or vote on his/her own protocol, but is allowed to remain in the meeting room while the protocol is being discussed. However, this may place undue pressure on colleagues regarding an objective and forthright discussion of the submitted protocol. It is hence important for RECs to implement a policy that ensures that scientific members who submit their protocols to the RECs that they themselves are members of leave the meeting room for the full duration of the discussion of their protocol.

While training varied widely amongst RECs, on average 54% of REC members had received training in research ethics or GCP. Surprisingly, only 20% of members had research ethics training on 75% of the 89 IRBs in the United States survey [25]. Definite and urgent training needs exist at some institutions.

A similar variability was reflected in terms of differences in workload, review time, infrastructure and training needs with historically disadvantaged RECs being more affected than historically advantaged RECs. This is a source of concern as historically disadvantaged RECs could potentially be further discriminated against in terms of accreditation criteria established by the NHREC. The health research ethics guideline released by the Department of Health [1] defines level 1 and 2 RECs in section 3.3.2. Level one RECs will be able to review research that poses minimal risk to human participants. These include health research proposals that that do not involve drug research, biomedical research involving human tissues, high-budget research (more than R250 000/$38 461 per annum) and high-technology research, that is, invasive, radiological, radio-active and other research requiring substantial equipment. In addition, collaborative international health research, multi-centre studies, and long-term studies exceeding one year in duration, are not considered to be within the competence of a level 1 REC. Level 2 RECs may review all types of health research. Level 1 RECs are encouraged, according to the guideline, to develop their skills to a level 2 REC within a 5 year period. The guideline does not elaborate on how this can be achieved or on what resources will be made available for training and development. Given the current situation with RECs in South Africa it is most likely that some historically disadvantaged RECs will only qualify for level one accreditation. While it is important to delineate RECs in terms of competence in order to maintain the highest standards of health research in SA, in the absence of redress aimed at raising the level of functioning of RECs at historically disadvantaged institutions, the NHREC system of accreditation will unintentionally serve to perpetuate the injustices of apartheid.

There are other consequences to the disparity in REC functioning. Most RECs review the research that is conducted at their own institutions. From the data on workload at the various RECs, it is evident that historically disadvantaged RECs already have very little research activity at their institutions. This will be reduced even further by an accreditation system that discriminates against disadvantaged institutions. It is established that research is a major source of funding for institutions and investigators alike. Hence low levels of research activity at historically disadvantaged institutions will impact on the ability of those institutions to build research funds both at an institutional level and at individual investigator level.

This study has conducted a superficial assessment of operations of health RECs and a more detailed assessment of review procedures would provide valuable data. Only the major RECs that review health research have been included in this survey. Research on smaller RECs attached to other training institutions is under way. The timing of this survey reflects the status quo in 2003 when the National Health Act and revised national guidelines were not developed. It will be important to survey the RECs again in 5–10 years to assess the impact of the new regulatory and legal mechanisms governing research ethics in SA.

There is enormous potential for future research on RECs in SA. Clearly training needs of REC members should to be assessed and addressed in more detail. Funding mechanisms for RECs should also be explored to find more sustainable and equitable means of funding RECs. Community perceptions in respect of demographic representation of investigators and RECs is an interesting and relevant area of further study. Finally, it would be important to survey other RECs not primarily involved in health research.

## Conclusion

Most health RECs surveyed are well established given the context of resource constraints that exist in SA and other developing countries. However, diversity in terms of race, gender and occupational identity is suboptimal. Variability in operations and training amongst RECs is a reflection of apartheid entrenched influences in tertiary education in SA. While legislation now exists to enforce standardization of research ethics review systems, no provision has been made for resources or capacity development, especially in historically disadvantaged institutions. This has the potential to perpetuate, if not increase the gap between highly efficient well resourced RECs and poorly resourced RECs in South Africa.

## Competing interests

The author(s) declare that they have no competing interests.

## Authors' contributions

KM conceived of the study, conducted all interviews with REC chairs and drafted the manuscript. LM conducted the data analysis and assisted with drafting of the manuscript. Both authors read and approved the final manuscript.

## Pre-publication history

The pre-publication history for this paper can be accessed here:


